# A Comparative Evaluation of the Cleaning Efficacy of Five Different Root Canal Irrigation Devices: A Histological Study

**DOI:** 10.1055/s-0043-1774325

**Published:** 2023-11-23

**Authors:** Alper Akçay, Melahat Gorduysus, Mehmet Omer Gorduysus, Lovely M. Annamma, Sevda Müftüoglu

**Affiliations:** 1 Department of Endodontics, Faculty of Dentistry, Hacettepe University, Ankara, Turkiye; 2 Apollon Dental Center, Antalya, Turkiye; 3 Department of Preventive and Restorative Dentistry, College of Dental Medicine, University of Sharjah, Sharjah, United Arab Emirates; 4 Department of Clinical Sciences, College of Dentistry, Ajman University, Ajman, United Arab Emirates; 5 Center of Medical and Bio-Allied Health Sciences Research, Ajman University, Ajman, United Arab Emirates; 6 Department of Histology and Embryology, Faculty of Medicine, Hacettepe University, Ankara, Turkiye

**Keywords:** SonicMax, RinsEndo, EndoVac, ultrasonic, histology, various irrigation devices

## Abstract

**Objectives** This study is aimed to evaluate the cleaning efficacy of five different irrigation systems as SonicMax, RinsEndo, EndoVac, passive ultrasonic irrigation (PUI), and manual needle irrigation (MNI) to histologically evaluate the presence of organic structures and the penetration of irrigation solution.

**Materials and Methods** Forty-two single-rooted, extracted human mandibular premolars were used in the study. Each tooth was decoronated at the cementoenamel junction and the root canals were instrumented using ProTaper rotary instruments in a crown-down manner. The specimens were randomly divided into five experimental groups ( *n* = 7) Group (1) SonicMax, group (2) RinsEndo,group (3) EndoVac, group (4) PUI, group (5) MNI, and the control groups ( *n* = 7). Each system used 2.5% sodium hypochlorite (NaOCl), 17% ethylenediamine tetraacetic acid (EDTA), and 2.5% NaOCl, respectively, in the experimental groups. The control group did not receive any final irrigation.

The measurements were analyzed by employing two-way analysis of variance multivariate results to show significant differences between the length of the dentin tubules in the apical, middle, and coronal of the six groups. The post-hoc test was used when groups were compared by pairs.

**Results** The results of this study indicate that among the five groups, the RinsEndo and EndoVac were found to be most effective in the cleaning of root canals. The RinsEndo shows highly significant results in the cleaning efficiency of the coronal and middle parts compared with the other groups. The cleaning efficiency in the apical area was the same for RinsEndo and EndoVac.

**Conclusion** The result of our study indicates that RinsEndo and EndoVac may be more effective in clinical practice.

## Introduction

The rationale of root canal treatment is to completely eradicate bacteria from diseased root canals to avoid reinfection during and after the procedure and restore healthy periapical tissue. 1 The success of a root canal depends on the chemicomechanical preparation of the root canal to eliminate all microorganisms. The root canal system is complicated, as it consists of existing isthmuses, anastomoses, and irregularities. Hence, the cleansing efficiency depends on numerous factors such as the type of solution, chemical effectiveness, concentration, temperature, exposure time, volume, contact time, and freshness of the irrigants used. Additionally, the technique used to deliver and activate the solution to the root canal is as important as the type of solution used. Furthermore, the complex structure of the root canals and the curvature along with the width of the root canal are also key factors. 2 3 Root canal irrigants remove debris and the smear layer formed during mechanical instrumentation in the root canal. The irrigants also remove biofilm, microorganisms, and their byproducts. The purpose of irrigations in root canal treatment is to eliminate all organic and inorganic remnants. 4

The proper irrigation method allows the irrigants to contact all parts of the root canal without apical extrusion into the periapical tissues. 5 Among the irrigation solutions, sodium hypochlorite (NaOCl) and ethylenediamine tetraacetic acid (EDTA) have been proven to remove debris and smear layers, respectively. The use of irrigants along with the activation systems has been observed to be more effective than the use of irrigant solutions alone. 6 7 The various irrigation systems commonly used and observed are the manual needle irrigation technique (MNI), passive ultrasonic irrigation (PUI) RinsEndo, SonicMax, and the EndoVac. 7 8 9 Many other systems are also available in the market yet the commonly employed systems are used in our research. Many studies have shown that the use of activated systems along with antimicrobial irrigation has improved healing properties when done on an infected tooth with abscess while observing through radiographic examination. 8 9 10 11

Several novel techniques and devices have been introduced to increase the effectiveness of irrigation. The most common irrigation technique is MNI, which delivers the irrigant into the root canal system using a syringe and a needle. It has been reported that the mechanical washing effect of the MNI system is insufficient to completely clean the root canal walls as it forms a smear layer. The efficiency of penetration of irrigants in small or curved canals is compromised and the smear layer is incompletely removed. The remaining smear layer consists of infective organic and inorganic substances that can be the source of postoperative infections. 12 Among the root canal preparation techniques, the ProTaper system is reported to be efficient in negotiating through curved canals and has a better centering ability compared with wave systems. 13 14 Hence, our study used the ProTaper system for the preparation of the root canals.

Our study was designed to compare the irrigation effect with five different activation techniques with a control group. This histologic study aimed to evaluate the cleaning efficacy of the group (1) SonicMax, group (2) RinsEndo, group (3) EndoVac, group (4) PUI, and group (5) MNI in comparison to a control group at the coronal, middle, and apical areas.

## Materials and Methods

### Selection and Preparation of Teeth

The inclusion criteria were 42 single-rooted, freshly extracted human mandibular premolars with fully formed apices. The tooth specimens that had fractures or resorption with abnormal root morphology were excluded from the study. The tooth selection for the experiment was the existence of a single root canal. A single canal was confirmed with the use of two digital radiographs taken in the buccal and proximal orientations. The debris and soft tissue pieces were removed. The teeth were then immersed in the physiological saline solution and kept there until needed. Each tooth was decoronated at the cementoenamel junction using a diamond disc. The size 10 K-file (Dentsply Maillefer, Ballaigues, Switzerland) was placed into the root canal until it could be seen in the apical foramen of the root, and the working length (WL) was established 1 mm short of this length. The root canals were instrumented using ProTaper (Dentsply-Maillefer Ballaigues, Switzerland) rotary instruments in a crown-down manner (Sequence-S1-Sx-S2-F1-F2-F3-F4) according to the manufacturer's recommendations. A size 10 K file was used to check the apical patency. Between each instrumentation, the canals were irrigated for 20 seconds with 1 mL of 2.5% NaOCl with a syringe and a 27-gauge needle. The needle was inserted into the canal until a slight resistance was encountered.

### Final İrrigation of the Root Canals

The specimens were randomly divided into five experimental ( *n* = 7) SonicMax, RinsEndo, MNI, PUI, EndoVac, and control groups ( *n* = 7). In the experimental groups, the last irrigation was done with 2.5% NaOCl, 17% EDTA, and 2.5% NaOCl, respectively, 5mL each. Lastly, a solution of 5 mL of saline was used as an irrigant to remove the residual solution remaining within the canal. After the last irrigation paper points were used to dry the root canals. To standardize the procedure, all specimens were prepared by the same operator under standardized conditions. The control group did not receive any final irrigation.

### Irrigation Techniques and Materials Used in This Study

*SonicMax* (Maximum Dental Inc., Secaucus, New Jersey, United States): SonicMax was used with 15 sizes of a K-file to activate the irrigation solution. The file was centered without making contact with the root canal wall and placed into the canal 1 mm shorter than the WL.
*RinsEndo* (Dürr Dental GmbH & Co KG, Bietigheim-Bissingen, Germany): RinsEndo was used with its needle (size 45 with a lateral opening of 7 mm). The delivery rate was arranged at 6.2 mL/min and was positioned in the coronal third of the canal. It was maintained without binding during the irrigation procedure based on the manufacturer's instructions.
*EndoVac* (Discus Dental, Culver City, California, United States): The Master delivery tip was placed into the coronal part. First, the macrocannula was used during the transferring of irrigation by moving constantly up and down; then in the same manner, the microcannula was applied into the canal 1 mm shorter than the WL, by using a 1 to 2 mm up and down motion.
***PUI:*** An ultrasonic device (Suprasson Pmax Satelec, Acteon, Marignac, France) was used with a Size 15 file (Irrisafe K 15 Satalec, Marignac, France). The Irrisafe was placed 1 mm shorter than the WL and activated at a power setting 5. The file was positioned in the center of the canal without contact.
*MNI****:*** Endo-Eze Needle (Endo-Eze, Ultradent, South Jordan, Utah, United States) was used: This is a 27-G side-vented needle, and it was placed into the canal 2 mm shorter than the WL without any binding. During the transfer of the irrigation solution to the root canal, the needle was moved 1 to 3 mm up and downwards.

### Preparation of Samples for Histological Evaluation

The roots were split into two halves longitudinally, and one-half of the tooth roots were prepared for histological evaluation for each root. To get histological sections, the samples obtained in each group were softened by decalcification in Decastro solution (ethanol: 300 mL [100%], distilled water: 670 mL, chloral hydrate: 50 g, nitric acid: 30 mL). The decalcification times were different and varied between 15 and 35 days on average. The solution containing the teeth was renewed every 3 days. The decalcified teeth were placed in formaldehyde, then placed in a tissue tracking device (Leica TP 1020, Leica Microsystems, Wetzlar, Germany), and left overnight. Paraffin blocks were prepared by embedding the teeth in paraffin. The prepared blocks were cut 2 to 3μm thick in a microtome (Leica RM2255, Leica Microsystems, Wetzlar, Germany), deparaffinized, and stained with Masson's trichrome and hematoxylin-eosin. In hematoxylin-eosin staining, the samples were dried and kept in xylol for 45 minutes, then covered with Canada balsam on the slides. In Masson's trichrome staining, it was kept in xylol for at least 10 and to a maximum of 15 minutes, after which the slides were covered with Canada balsam.

**Fig. 1:**
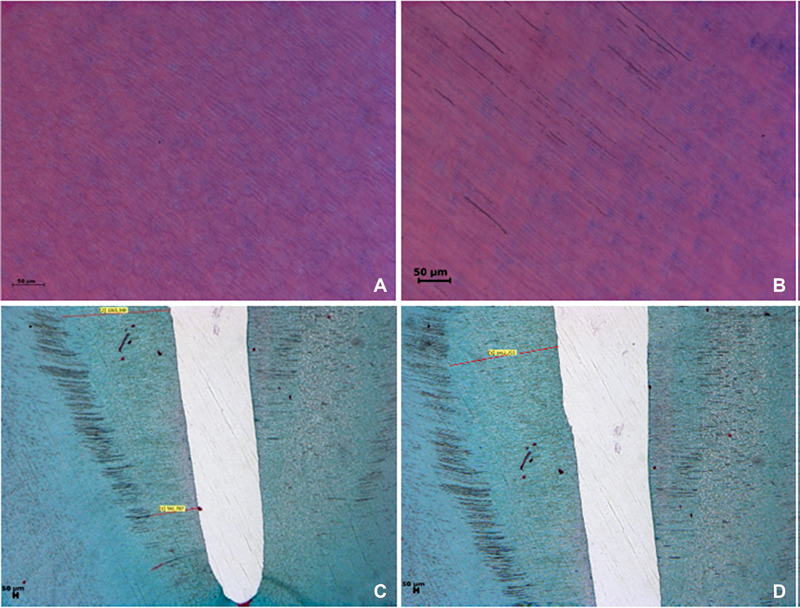
In histologic evaluation of ( **A** ) clean dentin tubules, ( **B** ) unclean dentin tübüller, ( **C** ) measurement of apical and middle third of the root canal dentin, and ( **D** ) measurement of the coronal part.

The presence of organic structures in the dentin surface and tubules along with the penetration depth of the applied irrigation solution were investigated. For this purpose, the blocks examined under a light microscope (Leica DM 6000B, Leica Microsystems, Wetzlar, Germany) were photographed with a digital camera (Leica DC 490 digital camera Leica Microsystems, Wetzlar, Germany). Once the dentin tubules were viewed clearly from the canal lumen, the penetration distance was measured on the photograph. Measurements were made for the apical, middle, and coronal regions. The measurements obtained were analyzed statistically, on how much tubule penetration had occurred along with the cleansing efficiency for all groups. The apical, middle, and coronal root parts of various irrigation techniques were compared ( Fig. 1 ) by the measurement method made with tubules viewed as clean and unclean.

## Statistical Analysis

**Fig. 2:**
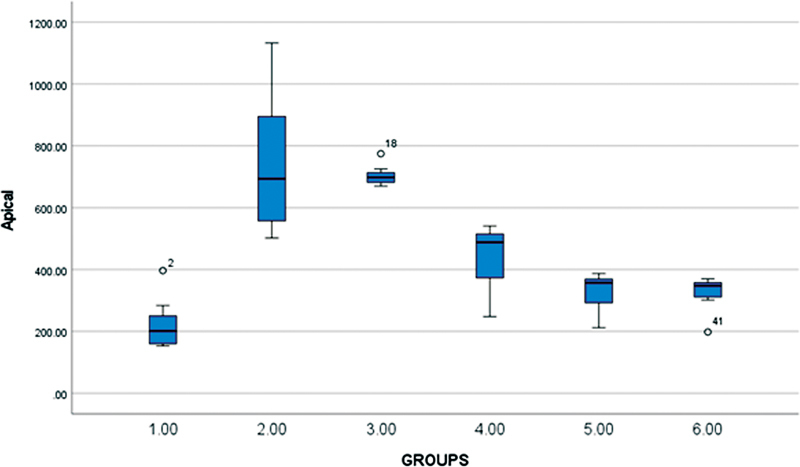
Comparative histogram for the apical portion of six groups. Outliers in whisker plot are either represented as 0 or asterisk.

**Fig. 3:**
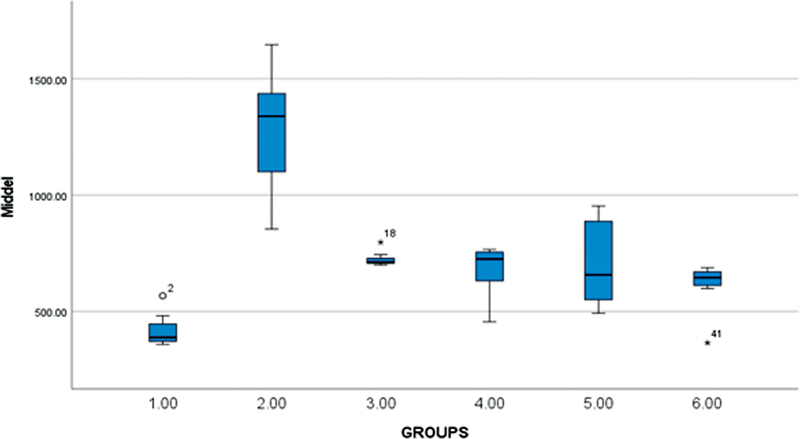
Comparative histogram for the middle portion of six groups. Outliers in whisker plot are either represented as 0 or asterisk.

**Fig. 4:**
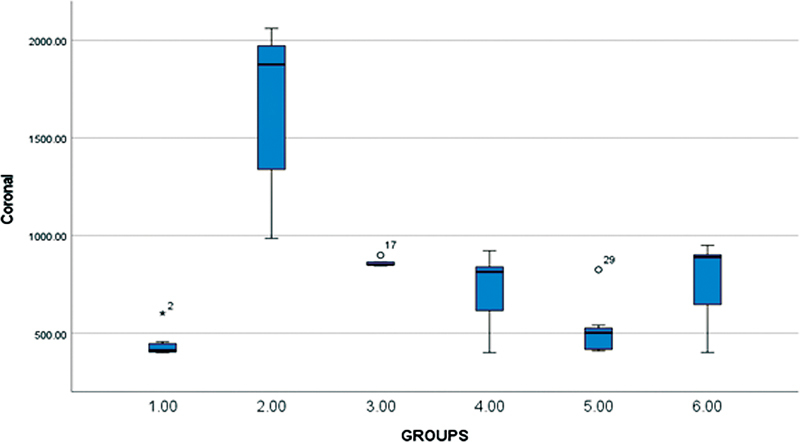
Comparative histogram for the coronal portion of six groups. Outliers in whisker plot are either represented as 0 or asterisk.

The obtained data were tabulated and statistically analyzed using (IBM SPSS statistics version 28.0.0.0) with a significant level fixed at 5% (α = 0.05). The data were analyzed to check the normality and by two-way analysis of variance (ANOVA) and the post-hoc test. Two-way ANOVA was used to compare and analyze the variance between the groups (Group 1 SonicMax, group 2 RinsEndo, group 3 EndoVac, group 4 PUI, group 5 MNI, and group 6 control) in the apical, middle, and coronal portion of the tooth. ( Table 1 ) The statistical significance was set at a *p* -value less than 0.05. ANOVA results show significant differences between the length of the dentin tubules in the apical, ( Fig. 2 ) middle, ( Fig. 3 ), and coronal ( Fig. 4 ) between the six groups. Hence, post-hoc testing was applied to reveal the differences between the means of six groups when the groups were compared by pairs.

**Table 1:** Test of normality comparison between groups

	Groups	Kolmogorov–Smirnov test a			Shapiro–Wilk test		
		Statistic	Df	Sig.	Statistic	Df	Sig.
Apical	G1 SonicMax	0.252	7	0.200	0.827	7	0.075
	G2 RinsEndo	0.217	7	0.200 *	0.876	7	0.210
	G3 EndoVac	0.256	7	0.182	0.874	7	0.203
	G4 Ultrasonic(PUI)	0.255	7	0.187	0.833	7	0.086
	G5 Manual(MNI)	0.263	7	0.153	0.883	7	0.239
	G6 Control	0.240	7	0.200 *	0.787	7	0.030
Middle	G1 SonicMax	0.269	7	0.137	0.823	7	0.069
	G2 RinsEndo	0.165	7	0.200 *	0.971	7	0.906
	G3 EndoVac	0.361	7	0.006	0.750	7	0.013
	G4 Ultrasonic(PUI)	0.294	7	0.067	0.797	7	0.038
	G5 Manual(MNI)	0.221	7	0.200 *	0.877	7	0.214
	G6 Control	0.321	7	0.028	0.704	7	0.004
Coronal	G1 SonicMax	0.293	7	0.070	0.685	7	0.003
	G2 RinsEndo	0.275	7	0.119	0.874	7	0.202
	G3 EndoVac	0.258	7	0.174	0.779	7	0.025
	G4 Ultrasonic(PUI)	0.376	7	0.003	0.755	7	0.014
	G5 Manual (MNI)	0.294	7	0.068	0.759	7	0.016
	G6 Control	0.357	7	0.007	0.725	7	0.007

Abbreviations: MNI, manual needle irrigation; PUI, passive ultrasonic irrigation.

* This is a lower bound of the true significance.

Lilliefors Significance Correction.

a Compares the cleaning efficiency between the groups at apical, Middle and coronal areas.

### Post-Hoc Tests

Tukey's honest significant difference post-hoc tests were performed to compare the apical, middle, and coronal with the mean difference ( Table 2 ) with group 2 RinsEndo followed by group 3 EndoVac showing better cleansing efficiency in the apical, middle, and coronal region.

**Table 2:** Mean comparison between groups by Tukey post-hoc test

Groups	Apical	Middle	Coronal
G1 SonicMax	224.5936	421.4720	445.0051
G2 RinsEndo	747.5820	1273.3711	1648.540
G3 EndoVac	704.8819	725.6524	860.3116
G4 Ultrasonic(PUI)	436.2746	674.2606	721.2277
G5 Manual(MNI)	325.6930	711.1659	517.8187
G6 Control	321.9946	608.9134	762.1601

Abbreviations: MNI, manual needle irrigation; PUI, passive ultrasonic irrigation.

## Results

The results of this study indicate that among the five groups, the group 2 RinsEndo shows highly significant results compared with the other groups in cleaning efficiency in the middle and coronal parts together. The cleaning efficiency in the apical area was the same for RinsEndo and EndoVac. Group 1 SonicMax showed the highest significance in the middle area and group 4 PUI is not significant in the coronal part but shows minimal significance in the apical and middle part. The MNI technique (group 5) and control (group 6) showed little significance in the apical part.

## Discussion

Irrigation of the root canal is critical for the success of root canal treatment. It is important that the irrigant is in contact with all root canal surfaces from coronal to apical and flushes out all the debris efficiently. The irrigant should function in a closed system with the flushed material exiting out coronally and not into periapical tissue or laterally. 15 The commonest irrigants used along with the activation system are NaOCI, EDTA, and chlorhexidine. 4 16 The tested activation systems in endodontics are only 6 out of the 13 activation systems. Of the various activation devices, ultrasonic was the most tested and reported followed by EndoVac. 17 18 Rödig et al compared three different groups and observed that RinsEndo was better overall than PUI and syringe irrigation. They also observed that if there are no irregularities in the canal, ultrasonic irrigation is more effective. 19 The apical third is the most compromised area in the irrigation process. 20

Many authors have stated that ultrasonic irrigation works better in apical areas than EndoVac. 21 22 Other authors found the EndoVac system to show better results as they observed that the bacterial numbers reduced after using EndoVac, especially in the infected tooth. 23 On the contrary, many authors have stated that there is no statistical difference in irrigation levels between EndoVac and PUI. 24 25 The SonicMax is a sonic-based activation device, while the PUI is a passive ultrasonic irrigation. Studies comparing these two have observed PUI is less effective, especially in the apical area than the sonic system, but the efficiency depends on the various depths from WL. 21 26 The endoactivator showed the best result for smear layer removal, but the apical smear layer scores were more than the middle and coronal even while using the different systems. 27 Kungwani et al compared EndoVac to conventional irrigation systems and observed by histology studies that EndoVac is superior in apical areas when compared with needle irrigation systems. 28 According to another study conducted by Akçay et al between various irrigation devices, the RinsEndo and EndoVac removed smear layer better and had minimum erosion effects on dentin as observed by Scanning electron microscope (SEM). 29 Many authors have contrary findings as PUI showed the least debris throughout the root compared with other systems as per SEM studies 30 Other authors recommended apical negative pressure irrigation to be superior compared with other techniques. 31 In our study, we compared five different activation groups (group 1 SonicMax, group 2 RinsEndo, group 3 EndoVac group 4 PUI group 5 MNI, and group 6 as controls). The coronal, middle, and apical cleaning efficacy was studied histologically. The observation from our histological study of all the experimental groups showed more cleaning efficiency than the control group. Among the experimental group, the EndoVac and RinsEndo systems were found to be more effective than the other irrigation systems in removing debris from the coronal, middle, and apical areas.

## Conclusion

The results of this study indicate that among the five groups, the RinsEndo and EndoVac were found to be most effective in the cleaning efficacy of root canals. The RinsEndo shows highly significant results compared with the other groups in the middle and coronal parts. The cleaning efficiency in the apical area was the same for RinsEndo and EndoVac. SonicMax showed the highest significance in the middle area and ultrasonic is not significant in the coronal part but shows minimal significance in the apical and middle parts. The MNI technique and control showed little significance in the apical part. The result of our study indicates that RinsEndo and EndoVac may be more effective in clinical practice. It is important to note that only the frequently used systems were compared and the result does not apply to other systems.
